# Chronic Otorrhœa; Caries of Petrous Bone; Fatal Meningitis

**Published:** 1878-10

**Authors:** F. C. Hotz

**Affiliations:** Chicago


*Chronic Otorrhœa; Caries of Petrous Bone; Fatal Meningitis.*

Although the number of cases of death from chronic suppuration of the middle ear is already pretty large, the publication of a short report of every new fatal case may not be quite useless, in view of the fact that physicians have but just begun to realize the great danger so often connected with that frequent and commonly neglected aural disease, chronic suppurative otitis.

On September 11, 1877, H. F., aged 42 years, peddler, called at my office. He has had a discharge from the left ear fifteen years, and never thought much of it, because the ear did not ache. But for the past three days he had been suffering the most agonizing pain in the left ear and around it, so that he could not sleep nor eat. I found the ear very sensitive; the post-auricular region slightly œdematous and tender; in the external meatus some pus; farther in, its lumen was blocked by a large, soft polypus, which, on removal with the wire snare, proved to be almost of the size of a cherry, and to have sprung from the promontory with a very thin peduncle. Membrana tympani and ossicles had completely disappeared.

My first impression was that the suffering of my patient was caused by retention of pus, whose free discharge through the external meatus was evidently impeded by the polypus. Therefore, I expected the removal of this growth would bring quick and perfect relief. The œdematous swelling behind the ear disappeared in a few days; but the pain persisted for two weeks; the suppuration did not abate to any appreciable degree, and a cautious examination with a probe of the place where the polypus originated, revealed extensive caries, and extreme sensitiveness of the bone. On the 1st of October the patient was free from pain, but complained of dizziness and a strange, heavy feeling in his head. Nevertheless he was determined to go into the country on some business. I tried to persuade him into a change of mind, because I apprehended serious complications. But he left the city on the next morning, and, as I was recently informed, died of meningitis two weeks afterwards.

CHICAGO, September, 1878. F. C. HOTZ.

---

VOMITING during anæsthesia produced by ether is so common an occurrence that we are liable to overlook the dangers which may attend it. A recent issue of the *British Medical Journal* contains the account of a lamentable case in which vomited matters found their way into the trachea and lungs and rapidly caused fatal asphyxia. The trachea was opened, but all efforts to save the patient's life proved futile. Suffocation from this cause is very rare, but it is well for us to be fore-warned of the possibility of such an accident.—*Cincinnati Lancet and Clinc.*

ETHER V. CHLOROFORM.—From the annual report of Prof. Bardeleben's clinic in Berlin, for the year 1876, we learn that death from chloroform occurred in that year four times among twelve to fifteen hundred narcoses. In all four cases a small amount of chloroform was used when death occurred. These accidents as well as an exceedingly large number of troublesome narcoses caused the professor to abandon chloroform and to use chloral-chloroform and ether. All narcoses since have been free from complications.

